# Global community science data on mammals underreport small and diurnal species

**DOI:** 10.1007/s10661-025-14654-7

**Published:** 2025-10-25

**Authors:** Lucas Rodriguez Forti, Judit K. Szabo

**Affiliations:** 1 Departamento de Biociências, Universidade Federal Rural Do Semi-Árido, Av. Francisco Mota, 572 - Bairro Costa E Silva, Mossoró , 59625-900 Rio Grande do Norte Brazil; 2 Research Institute for the Environment and Livelihoods, Charles Darwin University, Casuarina, NT 0909 Australia

**Keywords:** Biodiversity research, Community science, iNaturalist, Large datasets, Public attention, Mammalia

## Abstract

Although community (or citizen) science has revolutionized biodiversity data collection and expanded its potential application, these datasets are commonly affected by bias. For instance, observers’ attention towards biodiversity is often led by the aesthetic and economic values of organisms, resulting in the under- and overrepresentation of species. Mammals in general are more conspicuous and charismatic than most other groups and therefore hold a unique appeal for observers that are likely to contribute to community-science platforms. Nevertheless, not all mammals are equally attractive to the human observer, and depending on their ecological and phenotypical traits, different species are represented in varying degrees in datasets collected by non-professional scientists. Herein, we assess the contribution of community science observations to global mammal occurrence data, examining how species traits influence the number of contributed observations. We compiled and analyzed spatiotemporal patterns in over 2 million observations globally from the iNaturalist platform. We found that large, crepuscular, and widely distributed species were overrepresented compared to smaller, diurnal species with a narrower distribution. Marine mammals represented 3.1% of species and 7.0% of observations. Nevertheless, the average number of observations per species was 1217.2 for marine species compared to 690.5 for terrestrial species. While bats and rodents were underrepresented, less diverse groups such as elephants and monotremes were overrepresented. Around 55% of mammal species are currently represented in the iNaturalist dataset, and our findings reveal biases linked to species traits, offering opportunities to increase the representation of currently underrepresented mammal species in global biodiversity datasets by adaptive sampling.

## Introduction

Public participation in scientific endeavors has aided biodiversity research in reaching the global scale (Chandler et al., 2017). Millions of non-scientist members of the public are observing and recording nature around the planet, contributing a large amount of data to biodiversity repositories. In fact, the amount of data contributed to community-science platforms annually exceeds that collected by research institutions and opens novel avenues to explore macroecological and biogeographical questions (Della Rocca et al., 2024; La Sorte & Somveille, 2021; Mahecha et al., 2021). These findings could guide conservation actions, including those related to biodiversity loss and climate change (Devictor et al., 2010; McKinley et al., 2017), such as protected area establishment, species management, and the assessment of mitigation plans and threat status (Cranswick et al., 2022; Fontaine et al., 2022). Besides these benefits, engagement in biodiversity community science also increases public awareness of environmental issues (Forti, 2023; Forti & Szabo, 2024), further supporting species and ecosystem conservation (Forrester et al., 2017; Peter et al., 2021). Nevertheless, the usefulness of the resulting datasets is often limited by various types of bias. Besides the spatial and temporal biases that are also present in datasets collected by professionals (Jarrett et al., 2025; Sobral-Souza et al., 2024), unstructured biodiversity data collected by members of the public (i.e., non-professional scientists) using non-standardized methods are deeply affected by volunteer behavior, including the preference to observe particular (charismatic or threatened) species (Harvey et al., 2018; Tulloch & Szabo, 2012). Human attention towards biodiversity can be driven by the aesthetic and economic values of the given species (Mouquet et al., 2024), while distribution, rarity, and species traits have also been found to affect species detectability in different sampling schemes (Chatterjee et al., 2021; Schlossberg et al., 2018; Szabo et al., 2012). In general, community scientists have been found to prefer large, colorful, and widely distributed species compared to small and rare species (Callaghan et al., 2021a, 2021b; Forti, da Silva, et al., 2024; Forti, da Silva Passetti, et al., 2024; Stoudt et al., 2022). This is not a modern phenomenon; preference towards charismatic large African mammals has also been detected in historical sources from the sixteenth century (Monsarrat & Kerley, 2018).

However, at larger scales, abundance or distribution patterns are not strongly correlated with the number of species records in datasets collected by members of the public (Szabo et al., 2023). This pattern could be explained by volunteer observers ignoring common or “dull” species (Arazy & Malkinson, 2021; Arazy et al., 2024), while actively searching for species of interest, for instance, to complete their profiles in gamified platforms or when participating in mass events, such as Bioblitzes (Gigliotti et al., 2023; Meeus et al., 2023). Furthermore, during a Bioblitz, observations of the same organism can be submitted by different observers (Meeus et al., 2023), generating pseudoreplication that abnormally increases the representation of the given species in the dataset. In addition, the skill to find and record different species can be highly variable among observers (Callaghan et al., 2021a, 2021b; Kelling et al., 2015), and datasets collected by members of the public often overrepresent more conspicuous species compared to elusive, drab ones that require more skill to identify (Harvey et al., 2018) and are often erroneously identified (Gorleri et al., 2023). These factors introduce bias into datasets collected by non-professional scientists, which should be recognized and addressed before the data are used to infer occurrence patterns (Callaghan et al., 2019) and to guide conservation actions (Arazy & Malkinson, 2021; Deacon et al., 2023). Fortunately, the development and widespread adoption of statistical approaches for analyzing abundance patterns have greatly enhanced our ability to assess data quality, particularly with regard to the quantitative distribution of species (Magurran, 2004). A recent method for evaluating dataset integrity involves examining digit distribution patterns through the lens of Benford’s Law, which has demonstrated potential in identifying sampling heterogeneity in large-scale biodiversity datasets (Szabo et al., 2023). The application of Benford’s Law offers practical advantages, such as straightforward data preparation and ease of interpreting model fit results. By providing a mathematical expectation for digit frequencies in naturally occurring numerical datasets, it helps researchers identify potential anomalies or biases in data collection, making it an accessible and effective tool for assessing sampling heterogeneity and dataset integrity across a wide range of biological taxa.

Mammals, particularly those that have a large size and are vulnerable to extinction, are more appealing to humans than other organisms (Albert et al., 2018; Davies et al., 2018; Van Huynh, 2023). Tourism and human outdoor recreation activities can be highly influenced by the interest in observing mammals, especially focusing on large terrestrial (Kuenzi & McNeely, 2008; Ouboter et al., 2021) and marine (Mazzoldi et al., 2019) species. Nature-based tourism relies on people who love nature and frequent protected or other natural areas, and these visitors often contribute observations as community scientists (Procheş, 2024; Tulloch & Szabo, 2012). Mammals are a preferred target for volunteer observers who submit data to community-science platforms, even though this attention often presents challenges for conservation and can negatively affect vulnerable species (Ouboter et al., 2021; Ripple et al., 2017).

In this study, we quantify the contribution of community scientists to mammal occurrence data at the global scale, exploring the way species traits affect the number of observations of the given species. Depending on various species traits, people are more or less likely to record an observation of an individual animal (Arazy et al., 2024). Many mammal species are nocturnal, widely distributed, and larger than most other vertebrates (Hazlerigg & Tyler, 2019; Procko et al., 2023), while the overwhelming majority of non-scientist observers prefer to record species during the day (Forti et al., 2022; Kerley et al., 2003). Body size is also known to be a good proxy for charisma, i.e., larger species are generally considered more attractive to humans across different taxonomic groups (Berti et al., 2020). Therefore, we hypothesize that the number of observations of a given species is related to phenotypical and ecological traits and, in particular, we expect larger, diurnal, threatened, and more widely distributed mammal species to have more observations than smaller, exclusively nocturnal, non-threatened mammals with a narrower distribution.

## Materials and methods

We compiled data for all mammal species globally from iNaturalist (https://www.inaturalist.org/) using the GBIF repository (GBIF.org) on June 23, 2025 (10.15468/dl.8929n9). The available observations are all classified as Research Grade, i.e., have 2/3 consensus among the identifiers on the suggested species or subspecies. We recoded subspecies as their parent species after verifying species names and added the order they belong to using the taxonomy adopted by IUCN (2024). We excluded 71,640 observations of 13 domestic species. For each of the 3643 species, we collated data on trophic category (herbivore, insectivore, carnivore, or omnivore), activity (nocturnal, crepuscular, or diurnal), and habitat stratum (ground-dwelling, aerial, arboreal, scansorial, and marine) from Wilman et al. (2014), which is based on the widely used EltonTraits dataset. One species could belong to more than one category. We obtained individual body mass (in grams), total biomass (in grams), and estimated population size from Greenspoon et al. (2023). We obtained the biogeographic realm (Australian, Ethiopic, Nearctic, Neotropical, Oriental, Palearctic, or Marine) of the species from Holt et al. (2013), noting that one species could belong to more than one realm. We compiled global threat status data (IUCN, 2024) using the *rredlist* R package (Chamberlain, 2020). All statistical tests were performed in R version 4.2.1 (R Core Development Team, 2024). We calculated the number of observations per biogeographic realm to identify over- and underrepresented regions using QGIS v. 3.18.2 (QGIS Development Team, 2023). Using Benford’s Law, we tested the heterogeneity of the number of observations by species using the *benford* function of the *benford.analysis* package (Cinelli, 2014). This test is based on a natural law on the distribution of digits and allows testing whether the representation of each species follows a natural pattern of abundance (Szabo et al., 2023). We correlated the number of observations with the number of species of each order using the Spearman method through the *cor.test* R function. We used this correlation to check if species diversity in these groups was correlated with the number of observations. Using a goodness-of-fit (*χ*^2^) test, we compared the observed number of observations to the expected values, which were proportionally adjusted based on the number of species in each order.

In general, larger mammals are often active during the day, which may be linked to an improved physiological performance and predation avoidance (Gardezi & da Silva, 1999; van der Vinne et al., 2015). Therefore, before fitting a statistical model, we tested whether body mass differed between diurnal and non-diurnal species. We performed a Welch’s two-sample *t*-test to compare the means of two groups that have unequal variances. Diurnality was treated as a binary categorical factor (0, non-diurnal; 1, diurnal). The *t*-test was conducted to evaluate whether the mean body mass significantly differed between these two groups.

To account for the non-independence among species due to shared evolutionary history, we performed a phylogenetic ANOVA using the *phytools* package (Revell, 2012) in R. Species names were standardized to “genus and species” format and reconciled with the Open Tree of Life taxonomy using the *rotl* package (Michonneau et al., 2016). Unrecognized taxa and species with problematic Open Tree Taxonomy (OTT) identifiers were excluded from the analysis. A phylogenetic tree was constructed using the valid OTT IDs obtained, and branch lengths were assigned using Grafen’s method via the *compute.brlen*() function from the *ape* package (Paradis et al., 2004). We filtered the dataset to retain only species present in the phylogenetic tree and those with both body mass and diurnality data available. Similar to its treatment in the *t*-test, diurnality was treated as a binary categorical factor (0, non-diurnal; 1, diurnal), and body mass (g) was the response variable. The phylogenetic ANOVA was conducted using the *phylANOVA*() function in *phytools* with 1000 simulations (nsim = 1000), testing for statistically significant differences in body mass between diurnal and non-diurnal species, while incorporating phylogenetic covariance.

To evaluate whether the timing of species observations was consistent with their reported activity patterns, we first extracted the hour of each observation from the event timestamp in the occurrence dataset. For each species, we defined a categorical activity pattern based on the combination of three binary variables (diurnal, nocturnal, crepuscular) available in the trait dataset. Species were classified as “diurnal,” “nocturnal,” “crepuscular,” “cathemeral” (active both day and night, but not during crepuscular periods), “diurnal and crepuscular,” “nocturnal and crepuscular,” “flexible” (active in all periods), or “unknown.” We then joined the activity pattern information to each observation record by matching species names. Each observation was classified as occurring during the “diurnal” period (6:00–18:00), “nocturnal” period (18:00–6:00), or “crepuscular” period (defined as 5:00–7:00 and 17:00–19:00). An observation was considered “aligned’ if it occurred during a period that matched the species’ declared activity pattern: for example, a diurnal species observed during the day, a crepuscular species observed at dawn or dusk, a cathemeral species observed during the day or night (but not during crepuscular periods), and a flexible species observed at any time. For species with combined patterns (e.g., diurnal and crepuscular), alignment was defined as an observation occurring in any of the declared active periods. To assess the association between activity pattern and alignment statistically, we fitted a logistic regression model with alignment (aligned vs. not aligned) as the binary response variable and activity pattern as the predictor.

We fitted a linear mixed model using restricted (or residual) maximum likelihood (REML) and the nloptwrap optimizer, implemented in the *lme4* (Bates et al., 2015) and *lmerTest* (Kuznetsova et al., 2017) packages, to predict the effects of species traits on the number of observations per species. Order and occurrence in the Nearctic region were included as random effects to account for phylogenetic bias and regional differences, respectively. We controlled for regional differences in the model, as the number of observations was much higher in the Nearctic (1,231,760) compared to the other realms (Neotropical 791,609; Palearctic 556,796; Ethiopic 301,525; Australian 220,942; Oriental 188,809; Marine 128,126). Fixed effects included IUCN Red List status (Data Deficient, Least Concern, Near Threatened, Vulnerable, Endangered, and Critically Endangered), trophic category, habitat stratum, nocturnal/crepuscular/diurnal activity, estimated population size, body mass, total biomass, and geographical range. The last four variables were log-transformed due to their large range of values. Standardized parameters were obtained by fitting the model on a standardized version of the dataset (with rescaled variables). Model residuals were checked using qqnorm and qqline, and 95% confidence intervals (CIs) and *p*-values were computed using a Wald *t*-distribution approximation. Model quality was assessed using the *model_performance* and *check_singularity* functions of the *performance* package (Lüdecke et al., 2021), and collinearity was checked using the variance inflation factor (VIF) via the *check_collinearity* function.

## Results

**Fig. 1:**
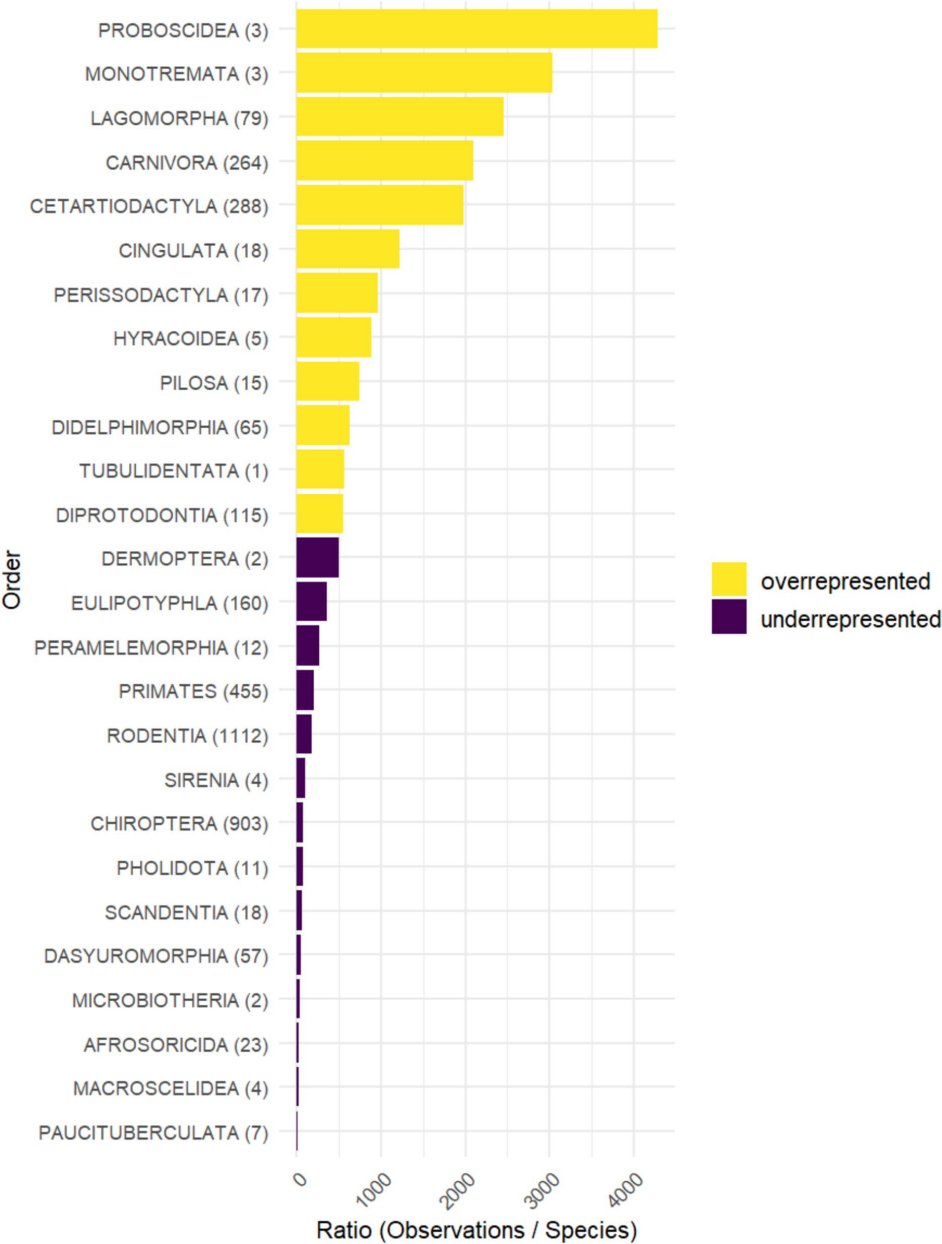
The number of Research Grade observations on the iNaturalist platform (as of July 2025) as a function of the number of species in each mammalian order (in parenthesis)

The dataset contained 1,921,231 research-grade observations of 3643 mammal species globally. White-tailed deer (*Odocoileus virginianus*), mule deer (*Odocoileus hemionus*), raccoon (*Procyon lotor*), eastern cottontail (*Sylvilagus floridanus*), coyote (*Canis latrans*), and red fox (*Vulpes vulpes*) were the most common species in the dataset, each with over 50,000 observations (for details see raw data in the online repository). On the other hand, 319 species were only represented by a single observation. The distribution of the number of observations among species did not conform to the distribution of digits expected by Benford’s Law (*χ*^2^ = 4096.4, df = 89, *p*-value < 2.2e-16; mean absolute deviation (MAD) 0.0080, distortion factor −13.51071; *N* = 3643). Even though the dataset was unbalanced, the number of observations among species was positively affected by the number of species in each order (*S* = 607.41, *p*-value = 1.407e-06, *ρ* = 0.79, *N* = 26). The observed number of observations for each order significantly deviated from the expected values, which were calculated based on a proportional distribution relative to species richness within each order (*X*^2^ = 3872686, df = 25, *p* < 2.2e-16). Based on the expected number of observations, 12 orders were overrepresented and 14 underrepresented (Fig. 1). The 112 marine species represented 3.1% of the species and 7% of the observations. In fact, the average number of observations per species was around double for marine mammals compared to terrestrial species, 1217.2 and 690.5, respectively.

Most observations (88.4%) were of species of Least Concern global conservation status, while Near Threatened and threatened (Vulnerable, Endangered, and Critically Endangered) species had only 3.86% and 7.32% of the observations, respectively. The remaining observations were Data Deficient (0.36%) or species that have not been evaluated by IUCN (0.04%). Half of the observations (50%) were of herbivores, followed by omnivores (23.4%), carnivores (16.8%), and insectivores (6.59%), while 3.11% of the observations were of species with no trophic information.

We also observed that most observations (1,589,296 or 82.7%) were of nocturnal species, followed by crepuscular (1,156,121 or 60.2%) and diurnal species (487,223.5 observations, 25.4%). Although diurnal species presented higher mean body mass values than non-diurnal species (32,030.8 ± 230,960.4 g and 7442.8 ± 71,630.6 g, respectively), phylogenetic ANOVA did not detect a statistically significant difference between the groups (*F* = 15.32, *p* = 0.727, based on 1000 simulations). The alignment between observation times and declared activity patterns varied substantially among categories. For strictly diurnal species, 76.8% of observations occurred during the expected diurnal period. Species classified as diurnal, crepuscular, and flexible also showed high alignment, with 84.9% and 100% of observations, respectively, matching their expected activity periods. In contrast, only 34.4% of observations for strictly nocturnal species were aligned with nocturnal hours, and this proportion was 39.0% for nocturnal and crepuscular species and 17.3% for crepuscular species. The logistic regression model testing the association between declared activity patterns and the alignment of observation times indicated that the activity pattern was a strong predictor of alignment (null deviance = 4,343,310; residual deviance = 3,275,258; AIC = 3,275,272). Most activity pattern categories showed statistically significant differences in alignment probability compared to the reference category (all *p*-values < 0.001), except for flexible species (*p* = 0.09).

Ground-dwelling species represented 78.4% of the observations, followed by marine, arboreal, scansorial, and aerial mammals (with 7%, 5.1%, 4.8%, and 1.7%, respectively), and 3% of observations were of species with no information about life style.

The fitted model to predict the number of observations based on species traits had a conditional *r*^2^ = 0.235. Fixed effects alone had marginal *r*^2^ = 0.071. The model showed that species with higher estimated populations had fewer observations (Table 1). The number of observations outside the Nearctic was significantly lower than within this realm (*β* = −1682.3, 95% CI [−2063.6, −1301.0], *t* = −8.6468, *p* = 1.065818e-17; Std. *β* = −194.56). Therefore, we controlled for this effect in the model presented in Table 1.

**Table 1:** Effects on the number of observations of mammal species on the iNaturalist platform based on a generalized linear mixed model (glmm) with order and occurrence in Nearctic region as random factors (*N* = 2108). The model presented AIC = 38,899.138; *R*^2^ = 0.235; ICC = 0.176; RMSE = 2577.1; and Sigma = 2592.94. The model did not presented singularity, and the variables had low collinearity (VIF < 3)

Fixed effects	Estimate	Std. error	2.5% CI	97.5% CI	df	*t* value	Pr(>|*t*|)
(Intercept)	640.612	1015.089	−1348.93	2630.15	2.118	0.631	0.589316
Data Deficient	902.167	487.792	−53.89	1858.22	2003.128	1.849	0.064534
Endangered	437.323	396.612	−340.02	1214.67	2078.360	1.103	0.270307
Least Concern	969.793	398.561	188.63	1750.96	1930.286	2.433	0.015055
Near Threatened	580.599	418.868	−240.37	1401.57	1986.632	1.386	0.165868
Vulnerable	576.139	394.334	−196.74	1349.02	2037.436	1.461	0.144158
Herbivore	13.713	310.432	−594.72	622.15	160.996	0.044	0.964820
Insectivore	147.834	328.675	−496.36	792.02	646.621	0.450	0.653013
Trophic Level Not Assigned	150.208	1339.606	−2475.37	2775.79	1935.627	0.112	0.910733
Omnivore	79.528	305.234	−518.72	677.78	362.250	0.261	0.794589
Arboreal	−155.073	295.133	−733.52	423.38	220.783	−0.525	0.599809
Ground	−209.897	288.192	−774.74	354.95	64.026	−0.728	0.469070
Marine	−444.296	2622.548	−5584.40	4695.80	2077.917	−0.169	0.865488
Scansorial	−37.308	325.886	−676.03	601.42	441.814	−0.114	0.908908
Nocturnal	−214.155	238.803	−682.20	253.89	1389.924	−0.897	0.369989
Crepuscular	447.719	179.578	95.75	799.69	1907.248	2.493	0.012745
Diurnal	−603.225	216.599	−1027.75	−178.70	1717.780	−2.785	0.005412
log_estimated_population	−265.462	81.722	−425.63	−105.29	1356.995	−3.248	0.001189
log_body_mass	700.607	100.260	504.10	897.11	83.420	6.988	6.32e-10
log_g_biomass	142.064	69.629	5.59	278.53	2085.998	2.040	0.041446
log_area	272.452	73.602	128.19	416.71	1765.825	3.702	0.000221

**Fig. 2:**
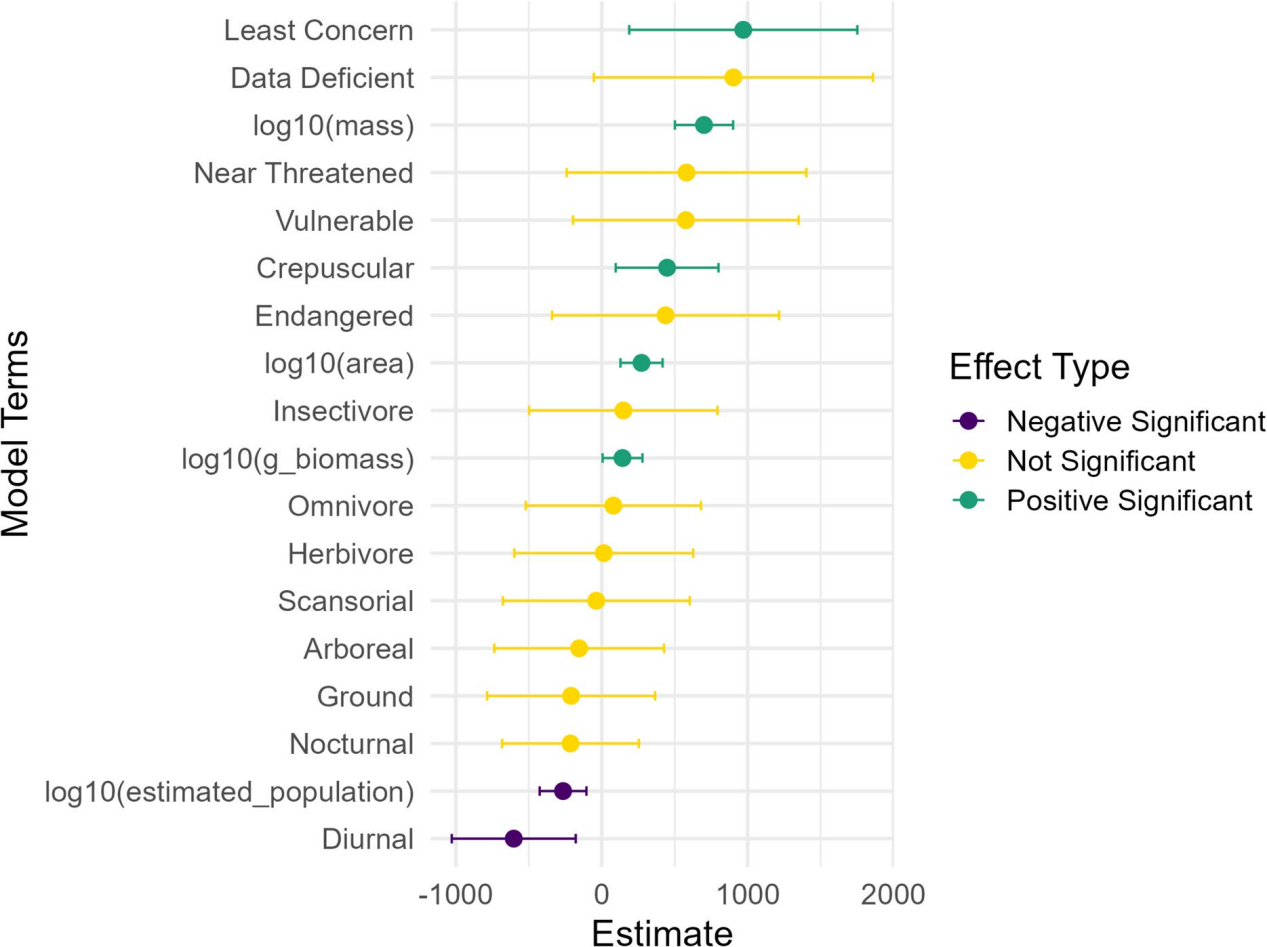
Forest plot showing the estimates and 95% confidence intervals for model terms when predicting the number of observations for 2108 mammal species at the global scale based on iNaturalist data. Points represent the estimated values, while horizontal bars indicate the confidence intervals. Green bars indicate a significant positive effect, where the confidence interval excludes zero, yellow indicates non-significant results, where the confidence interval includes zero and dark purple indicates significant negative results, where the confidence interval excludes zero. Note that we removed the values of intercept and marine species from the figure, as their effect was not significant and they had disproportionately high variability compared to the other terms

While Least Concern threat status, crepuscular habits, larger mass, larger total biomass, and larger area of occurrence positively affected the number of observations, diurnal habits and estimated population size had negative effects (Fig. 2).

## Discussion

At the time of writing this article, iNaturalist had observations of approximately 55% of the mammal species around the world. Biodiversity data in global marine and terrestrial data deposits are known to be biased, and the vast majority of the world is undersampled (Hughes et al., 2021). At a 1° resolution (~ 12,400 km^2^ at the equator), mammals have only been sampled in about half of the world, i.e., 52% of the oceans and 51% of terrestrial ecosystems (Hughes et al., 2021). While the spatial distribution of community scientists around the world is highly unbalanced (Tiago et al., 2017), which is known to contribute to the bias of global datasets (Hughes et al., 2021), community science has undoubtedly contributed to our knowledge of the distribution of mammal species on Earth. Our results show that species of a large body size, crepuscular habits, and wide distribution are represented in higher numbers in our global dataset compared to small, diurnal mammals with a restricted geographical distribution. Interestingly, more common species were proportionally less observed compared to rare species. These results did not follow our prediction based on Benford’s Law, as the data did not conform to the expected digit distribution based on the number of observations across species. High conformity would be expected if the number of observations were considered a proxy for species abundance (Szabo et al., 2023). This pattern could be caused by the lower representativeness of rodents and chiropterans, two highly diverse orders, compared to other groups in the dataset. Although these mammals are considered abundant, they move quickly (Wilson et al., 2015), are mostly small, and hide during the day (Arévalo et al., 2020), presumably making their non-directed (casual or incidental) observation more challenging. The distribution of body masses of the world’s mammal species is right-skewed on a logarithmic scale (Gardezi & da Silva, 1999), and Rodentia, Insectivora, and Chiroptera are particularly “light” orders. Besides their weight, their nocturnal habits, similar to some other taxonomic groups, such as Primates and Eulipotyphla (true shrews, moles, and hedgehogs), make these species more challenging to observe. It is not uncommon to find observations on iNaturalist that capture evidence of an animal’s activity—such as burrows, shelters, and footprints—rather than the animal itself (LRF unpublished data). Similarly, for more common species, observations of captive, trapped, or dead individuals are common, or they are of live, free-living mammals that are exploring human resources (LRF unpublished data). Identification to the genus or species level for these taxa—particularly Rodentia and Chiroptera—is challenging and often impossible without examining cranial, dental, or even genetic characteristics that are diagnostic for each group (Chornelia et al., 2022; Galan et al., 2012). Consequently, the lack of adequate resources for identifying cryptic species may lead to a biased distribution of records in which observations that should be attributed to multiple distinct species are instead grouped under a single name. This can result in an artificial inflation of occurrences for certain taxa, masking true species diversity and potentially distorting observed patterns (Gorleri & Areta, 2022). A simpler, alternative hypothesis, which does not negate the former, is that these shy, small, nocturnal species are not that attractive for humans, since many of them are seen as pests or vectors of important diseases (Rego et al., 2015; Salmón-Mulanovich et al., 2016).

Even though a large proportion of mammal species are nocturnal (Bennie et al., 2014), nocturnal species were rarely observed during the night in our dataset. Instead, most records of nocturnal and crepuscular species occurred outside their typical activity windows, during daylight hours. This pattern is consistent with previous findings that observer activity and detectability are much higher during the day (Di Cecco et al., 2021; Frey et al., 2017; Rowcliffe et al., 2014), leading to a strong bias in community-science datasets. In contrast, diurnal and flexible species are generally observed during their expected activity periods. Being diurnal in mammals may be an energy-saving strategy, as it reduces daily energy expenditure compared to nocturnal activity, particularly in species facing environmental challenges, such as food scarcity and temperature fluctuations (van der Vinne et al., 2015). This pattern is surprising, as we found that diurnal mammals tend to be larger than non-diurnal species (mean body mass 32,030.8 ± 230,960.4 g for diurnal species and 7442.8 ± 71,630.6 g for non-diurnal species). However, when accounting for shared evolutionary history, this difference was not statistically significant. Patterns of diurnality were also not evenly distributed across biogeographic regions, which can further bias observed trends (Bennie et al., 2014). Additionally, some mammals are known to shift their activity habits depending on environmental conditions; for instance, Cougars (*Puma concolor*) have been found to be less nocturnal in areas with higher road density (Procko et al., 2023). As an expected bias, species with a wide distribution range and a larger body size had more observations than spatially restricted, small species, potentially because the former were easier to record by a larger community of observers with variable detection skills. Similar results have been reported for birds—species with larger geographical distributions had a larger number of observations in several independent datasets (Forti, da Silva Passetti, et al., 2024). In addition, larger bird species are often more observed than small ones (Jayalath et al., 2023). Furthermore, the “Big Five” and a handful of other large and charismatic African mammals are preferentially reported in historic descriptions (Monsarrat & Kerley, 2018), are highly valued by tourists (Kerley et al., 2003; Maciejewski & Kerley, 2014), and are massively overrepresented in community-science platforms (Steger et al., 2017). In fact, Proboscidea and Carnivora were among the top three orders of the most overrepresented mammals in this study. Furthermore, body size is a powerful predictor of home range size in mammals (Tucker et al., 2014), and both properties can positively affect the number of observations in community-science datasets.

In the case of marine mammals, observations of 4% of genera that were observed within 5 km of the coast make up 66% of the records in global marine biodiversity data (Hughes et al., 2021). Surveying complex (i.e., three-dimensional) marine environments is logistically difficult and has a high financial cost, and cetaceans and other marine mammals are often elusive and migratory, further complicating their observation (Harvey et al., 2018). Compared to the traditional surveys, community scientists observed more Humpback (*Megaptera novaeangliae*) and Killer Whales (*Orcinus orca*), which are often conspicuous, and fewer Minke Whales (*Balaenoptera* spp.), which are more elusive and require more skill to identify (Harvey et al., 2018). For terrestrial species, sampling is more even but is closely associated with cities, with 22% of mammal records found within 1 km of cities (Hughes et al., 2021). Even within cities, charismatic species are usually overrepresented due to their high intrinsic values, as seen in the case of Red Foxes (*Vulpes vulpes*) and European Hedgehogs (*Erinaceus europaeus*) in a UK study (Boakes et al., 2024).

Least Concern and Data Deficient status positively affected the number of observations of a species in the iNaturalist dataset, while being classified as threatened had no effect. In general, species classified as Least Concern typically have stable or increasing populations and occupy broad geographic ranges (IUCN Standards & Petitions Committee, 2022)—traits that contribute to increasing the number of observations in datasets collected by community scientists. On the other hand, a set of factors that make a species less attractive to humans include rarity (a common trait of threatened species, also described to have a negative effect on the number of observations for birds—see Forti et al., 2024a, 2024b), and the difficulty of observing small and fast-moving species, particularly bats and rodents, which are also less attractive to humans (Luciano et al., 2022; Small, 2012).

## Conclusions

Our results highlight certain biases related to species traits that can be addressed to improve representation of mammals in global biodiversity datasets. First of all, the 13 underrepresented orders deserve more attention. The preference for charismatic and easy-to-observe species is also known to plague species-specific donations (Walpole & Leader-Williams, 2002) and scientific projects (Fontaine et al., 2022). Addressing spatial and temporal biases in the global iNaturalist dataset for mammals is essential and urgent to develop improved strategies for future adaptive sampling (Callaghan et al., 2022). Public participation in biodiversity research can produce better fruits for conservation, and our results can help to drive it to new horizons considering how species traits can be taken into account in future initiatives emerging to cover global dataset deficiencies.

## Data Availability

No datasets were generated or analysed during the current study.

## References

[CR1] Albert, C., Luque, G. M., & Courchamp, F. (2018). The twenty most charismatic species. *PLoS ONE,**13*(7), Article e0199149. 29985962 10.1371/journal.pone.0199149 PMC6037359

[CR2] Arazy, O., Kaplan-Mintz, K., Malkinson, D., & Nagar, Y. (2024). A local community on a global collective intelligence platform: A case study of individual preferences and collective bias in ecological citizen science. *PLoS ONE,**19*(8), Article e0308552. 10.1371/journal.pone.0308552 39186522 10.1371/journal.pone.0308552 PMC11346665

[CR3] Arazy, O., & Malkinson, D. (2021). A framework of observer-based biases in citizen science biodiversity monitoring: Semi-structuring unstructured biodiversity monitoring protocols. *Frontiers in Ecology and Evolution,**9*, 693602.

[CR4] Arévalo, R. L. M., Amador, L. I., Almeida, F. C., & Giannini, N. P. (2020). Evolution of body mass in bats: Insights from a large supermatrix phylogeny. *Journal of Mammalian Evolution,**27*, 123–138. 10.1007/s10914-018-9447-8

[CR5] Bates, D. M., Maechler, M., Bolker, B. M., & Walker, S. (2015). Fitting linear mixed-effects models using lme4. *Journal of Statistical Software,**67*(1), 1–48.

[CR6] Bennie, J. J., Duffy, J. P., Inger, R., & Gaston, K. J. (2014). Biogeography of time partitioning in mammals. *Proceedings of the National Academy of Sciences of the United States of America,**111*(38), 13727–13732. 10.1073/pnas.1216063110 25225371 10.1073/pnas.1216063110 PMC4183310

[CR7] Berti, E., Monsarrat, S., Munk, M., Jarvie, S., & Svenning, J.-C. (2020). Body size is a good proxy for vertebrate charisma. *Biological Conservation,**251*, Article 108790.

[CR8] Boakes, Z., Stafford, R., Bramer, I., Cvitanović, M., & Hardouin, E. A. (2024). The importance of urban areas in supporting vulnerable and endangered mammals. *Urban Ecosystems,**27*, 883–894. 10.1007/s11252-023-01492-z

[CR9] Callaghan, C. T., Bowler, D. E., Blowes, S. A., Chase, J. M., Lyons, M. B., & Pereira, H. M. (2022). Quantifying effort needed to estimate species diversity from citizen science data. *Ecosphere,**13*, e3966.

[CR10] Callaghan, C. T., Poore, A. G. B., Hofmann, M., Roberts, C. J., & Pereira, H. M. (2021). Large-bodied birds are over-represented in unstructured citizen science data. *Scientific Reports,**11*, 19073. 34561517 10.1038/s41598-021-98584-7 PMC8463711

[CR11] Callaghan, C. T., Poore, A. G. B., Mesaglio, T., Moles, A. T., Nakagawa, S., Roberts, C., Rowley, J. J. L., Vergés, A., Wilshire, J. H., & Cornwell, W. K. (2021). Three frontiers for the future of biodiversity research using citizen science data. *BioScience,**71*(1), 55–63. 10.1093/biosci/biaa131

[CR12] Callaghan, C. T., Rowley, J. J. L., Cornwell, W. K., Poore, A. G. B., & Major, R. E. (2019). Improving big citizen science data: Moving beyond haphazard sampling. *PLoS Biology,**17*(6), Article e3000357. 10.1371/journal.pbio.3000357 31246950 10.1371/journal.pbio.3000357 PMC6619805

[CR13] Chamberlain, S. (2020). *rredlist: ‘IUCN’ Red List client. R package version 0.7.0. *https://CRAN.R-project.org/package=rredlist. Accessed 18 Sept 2023.

[CR14] Chandler, M., See, L., Copas, K., Bonde, A. M. Z., López, B. C., Danielsen, F., Legind, J. K., Masinde, S., Miller-Rushing, A. J., Newman, G., Rosemartin, A., & Turak, E. (2017). Contribution of citizen science towards international biodiversity monitoring. *Biological Conservation,**213*, 280–294.

[CR15] Chatterjee, N., Schuttler, S. G., Nigam, P., & Habib, B. (2021). Deciphering the rarity-detectability continuum: Optimizing survey design for terrestrial mammalian community. *Ecosphere,**12*(9), Article e03748. 10.1002/ecs2.3748

[CR16] Chornelia, A., Lu, J., & Hughes, A. C. (2022). How to accurately delineate morphologically conserved taxa and diagnose their phenotypic disparities: Species delimitation in cryptic Rhinolophidae (Chiroptera). *Frontiers in Ecology and Evolution,**10*, Article 854509. 10.3389/fevo.2022.854509

[CR17] Cinelli, C. (2014). *benford.analysis: Benford analysis for data validation and forensic analytics. R package version 0.1.5. *https://www.rdocumentation.org/packages/benford.analysis/versions/0.1.5. Accessed 29 July 2025.

[CR18] Cranswick, A. S., Constantine, R., Hendriks, H., & Carroll, E. L. (2022). Social media and citizen science records are important for the management of rarely sighted whales. *Ocean and Coastal Management,**226*, Article 106271. 10.1016/j.ocecoaman.2022.106271

[CR19] Davies, T., Cowley, A., Bennie, J., Leyshon, C., Inger, R., Carter, C., Robinson, B., Duffy, J. P., Casalegno, S., Lambert, G., & Gaston, K. J. (2018). Popular interest in vertebrates does not reflect extinction risk and is associated with bias in conservation investment. *PLoS ONE,**13*(9), e0203694. 30256838 10.1371/journal.pone.0203694 PMC6157853

[CR20] Deacon, C., Govender, S., & Samways, M. J. (2023). Overcoming biases and identifying opportunities for citizen science to contribute more to global macroinvertebrate conservation. *Biodiversity and Conservation,**32*, 1789–1806. 10.1007/s10531-023-02595-x

[CR21] Della Rocca, F., Musiani, M., Galaverni, M., & Milanesi, P. (2024). Improving online citizen science platforms for biodiversity monitoring. *Journal of Biogeography,**51*, 2412–2423. 10.1111/jbi.15000

[CR22] Devictor, V., Whittaker, R. J., & Beltrame, C. (2010). Beyond scarcity: Citizen science programmes as useful tools for conservation biogeography. *Diversity and Distributions,**16*, 354–362.

[CR23] Di Cecco, G. J., Barve, V., Belitz, M. W., Stucky, B. J., Guralnick, R. P., & Hurlbert, A. H. (2021). Observing the observers: How participants contribute data to iNaturalist and implications for biodiversity science. *BioScience,**71*(11), 1179–1188.

[CR24] Fontaine, A., Simard, A., Brunet, N., & Elliott, K. H. (2022). Scientific contributions of citizen science applied to rare or threatened animals. *Conservation Biology*. 10.1111/cobi.13976 10.1111/cobi.13976 PMC10092489 35837961

[CR25] Forrester, T. D., Baker, M., Costello, R., Kays, R., Parsons, A. W., & McShea, W. J. (2017). Creating advocates for mammal conservation through citizen science. *Biological Conservation,**208*, 98–105.

[CR26] Forti, L. R. (2023). Students as citizen scientists: Project-based learning through the iNaturalist platform could provide useful biodiversity data. *Biodiversity,**24*(1–2), 76–78. 10.1080/14888386.2023.2174595

[CR27] Forti, L. R., da Silva, J. L. C., Ferreira, E. A., & Szabo, J. K. (2024). The implications of estimating rarity in Brazilian reptiles from GBIF data based on contributions from citizen science versus research institutions. *Integrative Conservation,**3*, 112–126. 10.1002/inc3.53

[CR28] Forti, L. R., da Silva Passetti, A. M. P. R., Oliveira, T., Lima, J., Queiros, A., Lopes, M. A. D. F., & Szabo, J. K. (2024). Global threat status, rarity, and species distribution affect prevalence of Atlantic Forest endemic birds in citizen-collected datasets. *Cambridge Prisms: Extinction,**2*, Article e17. 10.1017/ext.2024.22 40078798 10.1017/ext.2024.22 PMC11895707

[CR29] Forti, L. R., Retuci Pontes, M., Augusto-Alves, G., Martins, A., Hepp, F., & Szabo, J. K. (2022). Data collected by citizen scientists reveal the role of climate and phylogeny on the frequency of shelter types used by frogs across the Americas. *Zoology,**155*, 126052. 10.1016/j.zool.2022.126052 36152596 10.1016/j.zool.2022.126052

[CR30] Forti, L. R., & Szabo, J. K. (2024). Public collaboration to improve the future for science in Brazil. *Environmental Science & Policy,**162*, Article 103921. 10.1016/j.envsci.2024.103921

[CR31] Frey, S., Fisher, J. T., Burton, A. C., & Volpe, J. P. (2017). Investigating animal activity patterns and temporal niche partitioning using camera-trap data: Challenges and opportunities. *Remote Sensing in Ecology and Conservation,**3*(3), 123–132. 10.1002/rse2.60

[CR32] Galan, M., Pagès, M., & Cosson, J. F. (2012). Next-generation sequencing for rodent barcoding: Species identification from fresh, degraded and environmental samples. *PLoS ONE,**7*(11), Article e48374. 10.1371/journal.pone.0048374 23144869 10.1371/journal.pone.0048374 PMC3492341

[CR33] Gardezi, T., & da Silva, J. (1999). Diversity in relation to body size in mammals: A comparative study. *The American Naturalist,**153*(1), 110–123. 10.1086/303150 10.1086/303150 29578766

[CR34] Gigliotti, F. N., Franzem, T. P., & Ferguson, P. F. B. (2023). Rapid, recurring, structured survey versus bioblitz for generating biodiversity data and analysis with a multispecies abundance model. *Conservation Biology*. 10.1111/cobi.13996 10.1111/cobi.13996 36047702

[CR35] Gorleri, F. C., & Areta, J. I. (2022). Misidentifications in citizen science bias the phenological estimates of two hard-to-identify *Elaenia* flycatchers. *Ibis,**164*(1), 13–26.

[CR36] Gorleri, F. C., Jordan, E. A., Roesler, I., Monteleone, D., & Areta, J. I. (2023). Using photographic records to quantify accuracy of bird identifications in citizen science data. *Ibis,**165*(2), 458–471.

[CR37] Greenspoon, L., Krieger, E., Sender, R., Rosenberg, Y., Bar-On, Y. M., Moran, U., Antman, T., Meiri, S., Roll, U., Noor, E., & Ron, M. (2023). The global biomass of wild mammals. *PNAS*, *120*(10), e2204892120. 10.1073/pnas.2204892120 10.1073/pnas.2204892120 PMC10013851 36848563

[CR38] Harvey, G. K. A., Nelson, T. A., Paquet, P. C., Ferster, C. J., & Fox, C. H. (2018). Comparing citizen science reports and systematic surveys of marine mammal distributions and densities. *Biological Conservation,**226*, 92–100. 10.1016/j.biocon.2018.07.024

[CR39] Hazlerigg, D. G., & Tyler, N. J. C. (2019). Activity patterns in mammals: Circadian dominance challenged. *PLoS Biology,**17*(7), Article e3000360. 10.1371/journal.pbio.3000360 31306430 10.1371/journal.pbio.3000360 PMC6657935

[CR40] Holt, B. G., Lessard, J.-P., Borregaard, M. K., Fritz, S. A., Araújo, M. B., Dimitrov, D., Fabre, P.-H., Graham, C. H., Graves, G. R., Jønsson, K. A., Nogués-Bravo, D., Wang, Z., Whittaker, R. J., Fjeldså, J., & Rahbek, C. (2013). An update of Wallace’s zoogeographic regions of the world. *Science,**339*(74), 74–78. 23258408 10.1126/science.1228282

[CR41] Hughes, A. C., Orr, M. C., Ma, K., Costello, M. J., Waller, J., Provoost, P., Yang, Q., Zhu, C., & Qiao, H. (2021). Sampling biases shape our view of the natural world. *Ecography,**44*, 1259–1269.

[CR42] IUCN. (2024). *The IUCN Red List of threatened species. Version 2023–1. *https://www.iucnredlist.org*. *Accessed 2 Apr 2024.

[CR43] IUCN Standards and Petitions Committee. (2022). *Guidelines for using the IUCN Red List categories and criteria. Version 15.1. prepared by the standards and petitions committee. Downloadable from. *https://www.iucnredlist.org/documents/RedListGuidelines.pdf. Accessed 14 Sept 2023.

[CR44] Jarrett, D., Barnett, R., Bradfer-Lawrence, T., Froidevaux, J. S. P., Gibb, K., Guinet, P., Greenhalgh, J., Heath, B., Johnston, A., Lahoz Monfort, J. J., Rogers, A. D., Willis, S. G., & Metcalf, O. (2025). Mitigating bias in long-term terrestrial ecoacoustic studies. *Journal of Applied Ecology,**62*, 761–772. 10.1111/1365-2664.70000

[CR45] Jayalath, T. A., Lloyd-Smith, P., & Becker, M. (2023). Biodiversity benefits of birdwatching using citizen science data and individualized recreational demand models. *Environmental and Resource Economics,**86*, 83–107. 10.1007/s10640-023-00788-0

[CR46] Kelling, S., Johnston, A., Hochachka, W. M., Iliff, M., Fink, D., Gerbracht, J., Lagoze, C., La Sorte, F. A., Moore, T., Wiggins, A., Wong, W.-K., Wood, C., & Yu, J. (2015). Can observation skills of citizen scientists be estimated using species accumulation curves? *PLoS ONE,**10*(10), e0139600. 26451728 10.1371/journal.pone.0139600 PMC4599805

[CR47] Kerley, G. I. H., Geach, B. G. S., & Vial, C. (2003). Jumbos or bust: Do tourists’ perceptions lead to an under-appreciation of biodiversity? *South African Journal of Wildlife Research,**33*(1), 13–21.

[CR48] Kuenzi, C., & McNeely, J. A. (2008). Nature-based tourism. In O. Renn & K. D. Walker (Eds.), *Global risk governance. International Risk Governance Council Bookseries.* (Vol. 1, pp. 155–178.). Springer. 10.1007/978-1-4020-6799-0_8

[CR49] Kuznetsova, A., Brockhoff, P. B., & Christensen, R. H. B. (2017). LmerTest package: Tests in linear mixed effects models. *Journal of Statistical Software,**82*(13), 1–26.

[CR50] La Sorte, F. A., & Somveille, M. (2021). The island biogeography of the eBird citizen-science programme. *Journal of Biogeography,**48*, 628–638.

[CR51] Luciano, B. F., Elias, G. A., Zocche, J. J., Costa Neto, E. M., & Carvalho, F. R. (2022). The scientific literature on bats (Chiroptera) in Brazil: A scientometric analysis from 1954–2018. *Anais Da Academia Brasileira De Ciencias,**94*(4), e20211621. 10.1590/0001-3765202220211621 35830073 10.1590/0001-3765202220211621

[CR52] Lüdecke, D., Ben-Shachar, M. S., Patil, I., Waggoner, P., & Makowski, D. (2021). Performance: An R package for assessment, comparison and testing of statistical models. *Journal of Open Source Software,**6*(60), 3139. 10.21105/joss.03139

[CR53] Maciejewski, K., & Kerley, G. I. H. (2014). Understanding tourists’ preference for mammal species in private protected areas: Is there a case for extralimital species for ecotourism? *PLoS ONE,**9*(2), Article e88192. 10.1371/journal.pone.0088192 24505426 10.1371/journal.pone.0088192 PMC3914921

[CR54] Magurran, A. E. (2004). *Measuring biological diversity*. Blackwell. 10.1016/j.cub.2021.07.049 34637726

[CR55] Mahecha, M. D., Rzanny, M., Kraemer, G., Mäder, P., Seeland, M., & Wäldchen, J. (2021). Crowd-sourced plant occurrence data provide a reliable description of macroecological gradients. *Ecography,**44*(8), 1131–1142. 10.1111/ecog.05492

[CR56] Mazzoldi, C., Bearzi, G., Brito, C., Carvalho, I., Desiderà, E., Endrizzi, L., Freitas, L., Giacomello, E., Giovos, I., Guidetti, P., Ressurreiçao, A., Tull, M., & MacDiarmid, A. (2019). From sea monsters to charismatic megafauna: Changes in perception and use of large marine animals. *PLoS ONE,**14*(12), Article e0226810. 10.1371/journal.pone.0226810 31891944 10.1371/journal.pone.0226810 PMC6938407

[CR57] McKinley, D., Miller-Rushing, A. J., Ballard, H. L., Bonney, R., Brown, H., Cook-Patton, S. C., Evans, D. M., French, R. A., Parrish, J. K., Phillips, T. B., Ryan, S. F., Shanley, L. A., Shirk, J. L., Stepenuck, K. F., Weltzin, J. F., Wiggins, A., Boyle, O. D., Briggs, R. D., Chapin, F. S. I., & Soukup, M. A. (2017). Citizen science can improve conservation science, natural resource management, and environmental protection. *Biological Conservation,**208*, 15–28. 10.1016/j.biocon.2016.05.015

[CR58] Meeus, S., Silva-Rocha, I., Adriaens, T., Brown, P. M. J., Chartosia, N., Claramunt-López, B., Martinou, A. F., Pocock, M. J. O., Preda, C., Roy, H. E., Tricarico, E., & Groom, Q. J. (2023). More than a bit of fun: The multiple outcomes of a bioblitz. *BioScience,**73*(3), 168–181. 10.1093/biosci/biac100 36936381 10.1093/biosci/biac100 PMC10020829

[CR59] Michonneau, F., Brown, J. W., & Winter, D. J. (2016). rotl: An R package to interact with the Open Tree ofLife data. *Methods in Ecology and Evolution,**7*, 1476–1481. 10.1111/2041-210X.12593

[CR60] Monsarrat, S., & Kerley, G. I. H. (2018). Charismatic species of the past: Biases in reporting of large mammals in historical written sources. *Biological Conservation,**223*, 68–75. 10.1016/j.biocon.2018.04.036

[CR61] Mouquet, N., Langlois, J., Casajus, N., Auber, A., Flandrin, U., Guilhaumon, F., Loiseau, N., McLean, M., Receveur, A., Smith, R. D. S., & Mouillot, D. (2024). Low human interest for the most at-risk reef fishes worldwide. *Science Advances,**10*, eadj9510. 10.1126/sciadv.adj9510 39018399 10.1126/sciadv.adj9510 PMC466977

[CR62] Ouboter, D. A., Kadosoe, V. S., & Ouboter, P. E. (2021). Impact of ecotourism on abundance, diversity and activity patterns of medium-large terrestrial mammals at Brownsberg Nature Park, Suriname. *PLoS ONE,**16*(6), Article e0250390. 10.1371/journal.pone.0250390 34077471 10.1371/journal.pone.0250390 PMC8171955

[CR63] Paradis, E., Claude, J., & Strimmer, K. (2004). APE: Analyses of phylogenetics and evolution in R language. *Bioinformatics,**20*(2), 289–290. 10.1093/bioinformatics/btg412 14734327 10.1093/bioinformatics/btg412

[CR64] Peter, M., Diekötter, T., Höffler, T., & Kremer, K. (2021). Biodiversity citizen science: Outcomes for the participating citizens. *People and Nature,**3*, 294–311. 10.1002/pan3.10193

[CR65] Procheş, Ş. (2024). Nature observations between tourism, scientific data and pure appreciation. *Frontiers in Ecology and Evolution,**12*, Article 1417619. 10.3389/fevo.2024.1417619

[CR66] Procko, M., Naidoo, R., LeMay, V., & Burton, A. C. (2023). Human presence and infrastructure impact wildlife nocturnality differently across an assemblage of mammalian species. *PLoS ONE,**18*(5), Article e0286131. 10.1371/journal.pone.0286131 37228104 10.1371/journal.pone.0286131 PMC10212153

[CR67] QGIS Development Team. (2023). *QGIS geographic information system. Open source geospatial foundation project. *http://qgis.osgeo.org. Accessed 26 July 2025.

[CR68] R Core Development Team. (2024). *R: A language and environment for statistical computing. *(Foundation for Statistical Computing, Vienna, Austria). http://www.R-project.org/. Accessed 2 Feb 2024.

[CR69] Rego, K. M. D. C., Zeppelini, C. G., Lopez, L. C. S., & Alves, R. R. N. (2015). Assessing human-bat interactions around a protected area in northeastern Brazil. *Journal of Ethnobiology and Ethnomedicine,**11*, 1–8. 10.1186/s13002-015-0069-4 26576760 10.1186/s13002-015-0058-7 PMC4650336

[CR70] Revell, L. J. (2012). Phytools: An R package for phylogenetic comparative biology (and other things). *Methods in Ecology and Evolution,**3*(2), 217–223.

[CR71] Ripple, W. J., Wolf, C., Newsome, T. M., Hoffmann, M., Wirsing, A., & McCauley, D. J. (2017). Extinction risk is most acute for the world’s largest and smallest vertebrates. *Proceedings of the National Academy of Sciences of the United States of America, **114*(40), 10678–10683. 10.1073/pnas.1702078114 10.1073/pnas.1702078114 PMC5635868 28923917

[CR72] Rowcliffe, J. M., Kays, R., Kranstauber, B., Carbone, C., & Jansen, P. A. (2014). Quantifying levels of animal activity using camera trap data. *Methods in Ecology and Evolution,**5*(11), 1170–1179. 10.1111/2041-210X.12278

[CR73] Salmón-Mulanovich, G., Powell, A. R., Hartinger-Peña, S. M., Schwarz, L., Bausch, D. G., & Paz-Soldán, V. A. (2016). Community perceptions of health and rodent-borne diseases along the Inter-Oceanic Highway in Madre de Dios, Peru. *BMC Public Health,**16*, Article 755. 10.1186/s12889-016-3420-3 27506539 10.1186/s12889-016-3420-3 PMC4979164

[CR74] Schlossberg, S., Chase, M. J., & Griffin, C. R. (2018). Using species traits to predict detectability of animals on aerial surveys. *Ecological Applications,**28*(1), 106–118. 10.1002/eap.1632 28944528 10.1002/eap.1632

[CR75] Small, E. (2012). The new Noah’s ark: Beautiful and useful species only. Part 2. The chosen species. *Biodiversity,**13*(1), 37–53.

[CR76] Sobral-Souza, T., Silva Bosco, N., Candelária, L. P., Garcia, C. R., Layme, V. M. G., & Rodrigues, D. d. J. (2024). Spatial bias in sampling small rodents in the Atlantic Forest: A landscape and accessibility perspective. *Perspectives in Ecology and Conservation*, *22*, 297–305. 10.1016/j.pecon.2024.07.004

[CR77] Steger, C., Butt, B., & Hooten, M. B. (2017). Safari science: Assessing the reliability of citizen science data for wildlife surveys. *Journal of Applied Ecology,**54*(6), 2053–2062. 10.1111/1365-2664.12921

[CR78] Stoudt, S., Goldstein, B. R., & de Valpine, P. (2022). Identifying engaging bird species and traits with community science observations. *Proceedings of the National Academy of Sciences of the United States of America,**119*(16), e2110156119. 35412904 10.1073/pnas.2110156119 PMC9169790

[CR79] Szabo, J. K., Forti, L. R., & Callaghan, C. T. (2023). Large biodiversity datasets conform to Benford’s law: Implications for assessing sampling heterogeneity. *Biological Conservation,**280*, Article 109982. 10.1016/j.biocon.2023.109982

[CR80] Szabo, J. K., Fuller, R. A., & Possingham, H. P. (2012). A comparison of estimates of relative abundance from a weakly structured mass-participation bird atlas survey and a robustly designed monitoring scheme. *Ibis,**154*, 468–479.

[CR81] Tiago, P., Ceia-Hasse, A., Marques, T. A., Capinha, C., & Pereira, H. M. (2017). Spatial distribution of citizen science casuistic observations for different taxonomic groups. *Scientific Reports,**7*(1), 12832. 10.1038/s41598-017-13130-8 29038469 10.1038/s41598-017-13130-8 PMC5643322

[CR82] Tucker, M. A., Ord, T. J., & Rogers, T. L. (2014). Evolutionary predictors of mammalian home range size: Body mass, diet and the environment. *Global Ecology and Biogeography,**23*, 1105–1114. 10.1111/geb.12194

[CR83] Tulloch, A., & Szabo, J. K. (2012). A behavioural ecology approach to understand volunteer surveying for citizen science datasets. *Emu - Austral Ornithology,**112*, 313–325. 10.1071/MU12009

[CR84] van der Vinne, V., Gorter, J. A., Riede, S. J., & Hut, R. A. (2015). Diurnality as an energy-saving strategy: Energetic consequences of temporal niche switching in small mammals. *The Journal of Experimental Biology,**218*, 2585–2593. 10.1242/jeb.119354 26290592 10.1242/jeb.119354

[CR85] Van Huynh, A. (2023). Effect of IUCN Red List category on public attention to mammals. *Conservation Biology,**37*, Article e14050. 10.1111/cobi.14050 36661058 10.1111/cobi.14050

[CR86] Walpole, M. J., & Leader-Williams, N. (2002). Tourism and flagship species in conservation. *Biodiversity and Conservation,**11*(3), 543–547. 10.1023/a:1014864708777

[CR87] Wilman, H., Belmaker, J., Simpson, J., De La Rosa, C., Rivadeneira, M. M., & Jetz, W. (2014). Eltontraits 1.0: Species-level foraging attributes of the world’s birds and mammals. *Ecology,**95*(7), 2027.

[CR88] Wilson, R. S., Husak, J. F., Halsey, L. G., & Clemente, C. J. (2015). Predicting the movement speeds of animals in natural environments. *Integrative and Comparative Biology,**55*(6), 1125–1141. 10.1093/icb/icv106 26493609 10.1093/icb/icv106

